# Suppression of chorismate synthase, which is localized in chloroplasts and peroxisomes, results in abnormal flower development and anthocyanin reduction in petunia

**DOI:** 10.1038/s41598-020-67671-6

**Published:** 2020-07-02

**Authors:** Shiwei Zhong, Zeyu Chen, Jinyi Han, Huina Zhao, Juanxu Liu, Yixun Yu

**Affiliations:** 1 Guangdong Key Laboratory for Innovative Development and Utilization of Forest Plant Germplasm, College of Forestry and Landscape Architecture, South China Agricultural University, Guangzhou, 510642 China; 2 Lingnan Guangdong Laboratory of Modern Agriculture, Guangzhou, 510642 China; 3 College of Horticulture, South China Agricultural University, Guangzhou, 510642 China

**Keywords:** Plant sciences, Plant physiology

## Abstract

In plants, the shikimate pathway generally occurs in plastids and leads to the biosynthesis of aromatic amino acids. Chorismate synthase (CS) catalyses the last step of the conversion of 5-enolpyruvylshikimate 3-phosphate (EPSP) to chorismate, but the role of CS in the metabolism of higher plants has not been reported. In this study, we found that PhCS, which is encoded by a single-copy gene in petunia (*Petunia hybrida*), contains N-terminal plastidic transit peptides and peroxisomal targeting signals. Green fluorescent protein (GFP) fusion protein assays revealed that PhCS was localized in chloroplasts and, unexpectedly, in peroxisomes. Petunia plants with reduced PhCS activity were generated through virus-induced gene silencing and further characterized. *PhCS* silencing resulted in reduced CS activity, severe growth retardation, abnormal flower and leaf development and reduced levels of folate and pigments, including chlorophylls, carotenoids and anthocyanins. A widely targeted metabolomics analysis showed that most primary and secondary metabolites were significantly changed in pTRV2-PhCS-treated corollas. Overall, the results revealed a clear connection between primary and specialized metabolism related to the shikimate pathway in petunia.

## Introduction

**Figure 1:**
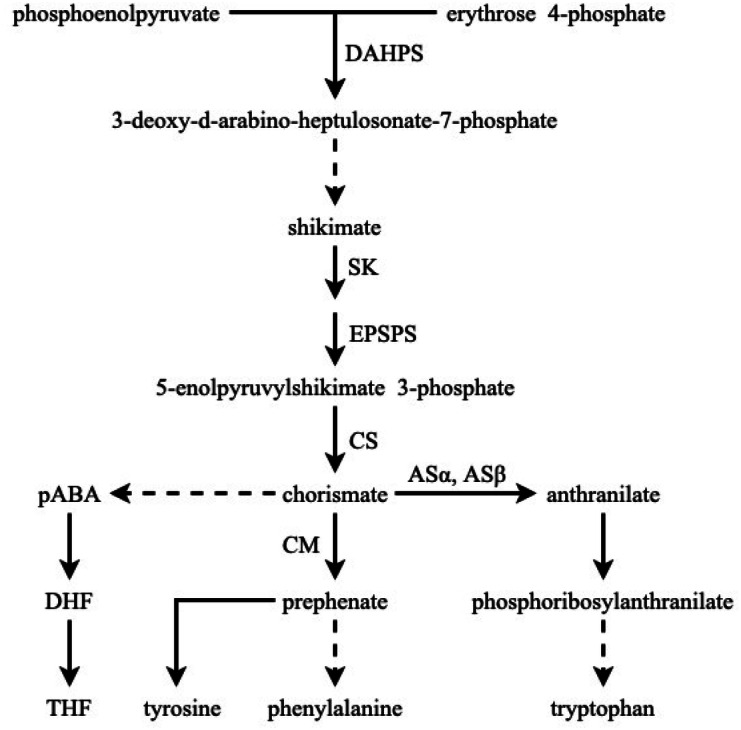
The shikimate pathway converts phosphoenolpyruvate and erythrose 4-phosphate to chorismate and aromatic amino acids in higher plants. Chorismate is an essential precursor in the major route of Tyr and Phe biosynthesis as well as in the other route of Tyr biosynthesis via anthranilate, which is catalysed by AS_α_ and AS_β_ using chorismate as the substrate. The dotted lines indicate more than one reaction, and the solid lines indicate only one step in the reaction. *AS* anthranilate synthase, *CM* chorismate mutase, *CS* chorismate synthase, *DAHPS* 3-deoxy-d-arabino-heptulosonate 7-phosphate synthase, *DHF* dihydrofolate, *ESPS* 3-phosphoshikimate 1-carboxyvinyltransferase, *p-ABA* para-aminobenzoic acid, *SK* shikimate kinase, *THF* tetrahydrofolate.

The shikimate pathway provides the basic building blocks for the synthesis of three aromatic amino acids as well as an array of aromatic secondary metabolites, such as flavonoids, alkaloids, and lignins. This pathway is highly conserved in fungi, bacteria, and plant species^1–3^ and consists of seven metabolic steps, and six key enzymes, including 3-deoxy-d-arabino-heptulosonate 7-phosphate synthase (DAHPS), 3-dehydroquinate synthase (DHQS), 3-dehydroquinate dehydratase (DHD)–shikimate dehydrogenase (SDH), shikimate kinase (SK), 5-enolpyruvylshikimate 3-phosphate (EPSP) synthase, and chorismate synthase (CS), are involved in these steps (Fig. 1).

Previous studies have indicated that many enzymes involved in the shikimate pathway have been found in plastids^2,4–12^. In addition, most shikimate pathway enzymes are synthesized as precursors with an N-terminal extension, which allows the post-translational uptake of the protein into chloroplasts^13,14^. In addition, the use of green fluorescent protein (GFP) fusion proteins or protein import assays has confirmed the plastidic localization of most enzymes in this pathway, such as DAHPS, anthranilate synthase, EPSPS, DHD/SDH, and SK, with the exception of CS^13–22^.

However, not all enzymes involved in the shikimate pathway are located in plastids. The isoforms of some enzymes involved in the shikimate pathway, such as DAHPS^23^ and EPSPS^4^, lack N-terminal transit peptides^24^. In addition, tobacco DHD/SDH2^17^ and chorismate mutase (CM) isoforms, denoted CM2^19,25^, in *Arabidopsis thaliana* and petunia have been shown to exhibit extraplastidic localization^17,19^.

Chorismate, the final product of the shikimate pathway, is subsequently formed by CS (CE 4.2.3.5), which catalyses the *trans*-1,4 elimination of phosphate from EPSP. A CS from a higher plant was first reported in 1986, and the activity of the enzyme was detected in tissue extracts and chloroplast preparations from pea (*Pisum sativum*)^6^. A *Corydalis sempervirens* CS was purified and characterized from a cell suspension culture, and a *CS* gene from a higher plant was first cloned^26^. *A. thaliana* possesses a single *CS* gene (At1g48850), whereas tomato possesses two differentially expressed *CS* genes, termed *LeCS1* and *LeCS2*^27^. Surveys of other species revealed that one to three *CS* genes were present in higher plants^28^. The *CS* genes in these species have a 5′ plastid import signal^28^. However, the role of CS in metabolism in higher plants has not been reported.

In this study, we characterized the single-copy *PhCS* gene in the petunia genome and analysed its localization. Surprisingly, an analysis using GFP fusion proteins revealed that PhCS was localized not only in chloroplasts but also in peroxisomes. Plants with reduced PhCS activity were generated using the virus-induced gene silencing (VIGS) approach. Suppression of PhCS resulted in abnormalities in the growth and development of leaves and flowers and reduction in the levels of anthocyanins and folate. A widely targeted metabolomics analysis showed that most primary and secondary metabolites in pTRV2-PhCS-treated corollas were significantly changed. Our findings demonstrate a clear connection between primary and specialized metabolism in petunia.

## Materials and methods

### Plant materials

Petunia ‘Ultra’ plants purchased from Sanli Horticultural Company of Guangzhou were grown under greenhouse conditions (23 ± 2 °C, 14-h light/10-h dark cycle, 60% relative humidity)^29^. The leaves, stems and roots were harvested at the vegetative stage when the height of the plants was approximately 25 cm. The flowers were collected at anthesis (corollas 90° reflexed) and immediately placed in tap water. Each 0.2-g tissue sample was wrapped in foil, frozen in liquid nitrogen and stored at – 80 °C until use^30^. All the experiments were conducted using three biological replicates from independently collected and extracted tissues unless otherwise noted.

### RNA extraction, RT-PCR and cloning of the petunia *PhCS* gene

Total RNA from the roots, stems, leaves and corollas was isolated using an RNA isolation kit (R4151-03, Magen, China) in accordance with the manufacturer’s recommendation. Reverse transcription of petunia mRNA was performed using an RNA reverse transcription kit (TSK314s, Tsingke Guangzhou, China) based on the manufacturer’s instructions. Full-length *PhCS* (Peaxi162Scf00747g00122.1) cDNAs were isolated using specific primers based on their sequences in the petunia genome (https://solgenomics.net/organism/Petunia_axillaris/genome).

### Sequence analysis

Multiple sequence alignments were performed, and a phylogenetic tree was generated using DNAMAN (version 5.2.2, Lynnon Biosoft, USA) software. An identity search for nucleotides and translated amino acids was conducted using the National Center for Biotechnology Information (NCBI) BLAST network server (https://blast.ncbi.nlm.nih.gov/Blast.cgi).

### Subcellular localization

The analysis of subcellular localization was performed according to a previously described protocol^31^. The pSAT-1403TZ vector (https://www.ncbi.nlm.nih.gov/nucleotide/56553541), which contains the GFP gene, and the pSAT-1450TZ vector (https://www.ncbi.nlm.nih.gov/nucleotide/56553574), which contains the RFP gene, were used for the construction of PhCS and fluorescent organelle marker constructs^32^. *PhCS* gene fragments were amplified by PCR and then cloned into the pSAT-1403TZ vector, in which the GFP fusions are driven by the CaMV 35S promoter. Sequence fragments of the mitochondrion-located marker gene encoding the first 29 aa of yeast ScCOX4^33,34^, the Golgi apparatus-located marker gene encoding the first 49 aa of GmMan1^35^ and the peroxisomal targeting sequence (PTS2)^36^ were generated by PCR and cloned into the pSAT-1450TZ vector, in which the RFP fusions are driven by the CaMV 35S promoter, to form the gRFP, mRFP and pRFP vectors, respectively, and visualized by confocal microscopy. All constructs were confirmed by sequencing. The primers used for PCR or overlap PCR are listed in Supplementary Table S1.

Petunia leaf protoplast isolation and poly(ethylene glycol)-mediated transfection were performed as described previously^37^. The fluorescence was analysed after 24 h of incubation in the dark. Confocal analyses were performed using a Zeiss (http://www.zeiss.com) LSM710 microscope. The excitation/emission wavelengths for GFP and RFP were 488/535 nm and 552/610 nm, respectively.

### Quantitative real-time PCR assays

Quantitative real-time PCR (qPCR) assays were performed according to previously described methods^31^. Briefly, to prepare each 25-μL reaction, 1 μL of cDNA was mixed with 12.5 μL of IQ SYBR Green Supermix (Bio-Rad Laboratories), 0.5 μL of forward primer, 0.5 μL of reverse primer, and 10.5 μL of sterile water. These samples were subjected to the following thermal cycling conditions: DNA polymerase activation for 4 min at 95 °C; 38 cycles of 45 s at 95 °C, 45 s at 52 °C or 55 °C, 45 s at 72 °C, and 45 s at 80 °C; and a final elongation step of 7 min at 72 °C. A melting curve was created by increasing the temperature at a rate of 0.5 °C every 10 s starting from 62 °C. The analyses were performed using two different cDNAs from the same time point (from two different RNAs), and each analysis was performed in triplicate. The amplicons were analysed by electrophoresis and sequenced once for identity confirmation. The quantification was based on an analysis of the threshold cycle (Ct) value as described by Pfaffl^38^. The analyses were conducted following the Minimum Information for Publication of Quantitative Real-Time PCR Experiments guidelines^29,39^. *Cyclophilin* (*CYP*) (accession no. EST883944) was used as the internal reference gene for quantification of cDNA abundance^40^. The data presented in the text represent the relative expression values calculated using *CYP*. The sequences of all the primers used in the qPCR analysis are described in Supplemental Table S2. Three biological replicates from each treatment were analysed.

### Agroinoculation of pTRV2 vectors

The 291-bp gene sequence of the 3′ untranslated region of *PhCS* was amplified by PCR using specific primers (Table S3), and the PCR products were inserted into the pTRV2 vector to form pTRV2-PhCS, which was used for suppression of PhCS. *Agrobacterium tumefaciens* (strain GV3101) cultures transformed with pTRV1 and pTRV2 or pTRV2-PhCS were prepared as previously described^29,41^. The *A. tumefaciens* culture was grown overnight at 28 °C in liquid YEP medium^42^ with 50 mg L^−1^ kanamycin and 200 μM acetosyringone, and *A. tumefaciens* cells were collected and resuspended in inoculation buffer containing 10 mM MgCl_2_, 10 mM MES (pH 5.5), and 200 μM acetosyringone to an OD_600_ of 10. After 3 h of incubation at 28 °C, bacteria containing pTRV1 were mixed with bacteria containing the pTRV2 or pTRV2 derivative at a ratio of 1:1. Approximately 300 μL of this mixture was then applied to the cut surface of 4-week-old petunia plantlets after removal of the apical meristems. Twenty-five to 30 plants were inoculated with each vector. The inoculated plants were grown under greenhouse conditions. Three biological replicates corresponding to three independent positive transformation events were included in this analysis.

### Measurement of the chlorophyll and carotenoid levels

The chlorophyll and carotenoid levels were measured according to a previously published protocol^43^. Fresh leaves (0.05 g) were shredded into filaments and submerged in 2 mL of DMSO for 3 h at 65 °C until the colour of the leaves turned from green to pale. Subsequently, 8 mL of 80% acetone diluted with weakly alkaline water was added to the DMSO, and the absorbance of the mixture was then measured at 663 nm, 646 nm, and 470 nm using a spectrophotometer (Shanghai Metash Instruments, V-5100B). All the experiments were conducted with five independent biological replicates. The chlorophyll content was quantified using the following equations: c (a) (mg/L) = 12.2 × A_663_ − 2.81 × A_646_; c (b) (mg/L) = 20.13 × A_646_ − 5.03 × A_663_; c (total) (mg/L) = c (a) + c (b); and c (caro) = (1,000 × A_470_ − 3.27 × c (a)   − 104 × c (b))/229. In addition, C = (m/0.1) × c; thus, the dilution factor was 0.1.

### Assays of CS activity

Total protein was extracted from 0.1 g of fresh corolla or leaf tissues of petunia using a Total Protein Extraction Kit (Jingkang, Shanghai, China, https://gelatins.com.cn/product-37158). The activity of CS was analysed using a Chorismate Synthase Activity Kit (Jingkang, Shanghai, China, https://gelatins.com.cn/product-42272). A standard curve was drawn with the following series of standard concentrations: 0 U/L, 25 U/L, 50 U/L, 100 U/L, 200 U/L, and 400 U/L. Prepared protein extracts were diluted five times and added to an ELISA plate with an HRP-labelled reagent, and the plate was then incubated for 60 min at 37 °C. All the liquid was subsequently discarded from the plate, and the plate was thoroughly washed with detergent to ensure that no liquid remained. A chromogenic agent was then added to the ELISA plate, and the plate was incubated in a dark environment for 15 min to allow the colour to change. After addition of the stop solution, the absorbance of the extracts was determined spectrophotometrically at 450 nm using a Varioskan LUX (Thermo Scientific, Waltham, MA, USA). Five independent biological replicates were included for each sample. More details are included in the specific instructions provided with the Chorismate Synthase Kit (Jingkang, Shanghai, China).

### Anthocyanin extraction and measurement

Anthocyanins were extracted from 0.2 g of finely ground petunia corollas as described previously^44,45^. Petunia corollas were extracted with 1 mL of acidic methanol containing 1% HCl (v/v) for 18 h at 25 °C. The plant materials were sedimented by centrifugation at 15,000*g* for 10 min, and 500 µL of the supernatant was mixed with 500 µL of Milli-Q H_2_O and 300 µL of chloroform in a 2.0 mL microcentrifuge tube. After centrifugation at 8,600*g* for 6 min, the supernatant (water–methanol phase) was transferred to a new tube. The absorption values of the extract were measured at A_530_ and A_657_ to determine the anthocyanin content using the formula A_530_ − 0.25A_657_, which allows subtraction of the chlorophyll interference. Three biological replicates of each treatment were analysed.

### Widely targeted metabolomics analysis

Petunia corollas were collected, freeze-dried and ground to powder, and 0.1 g of the powder was placed in 1.0 mL of 70% aqueous methanol at 4 °C overnight. The extract was then centrifuged at 10,000*g* for 10 min, and the supernatant was filtered through a microporous membrane (0.22-μm pore size) for liquid chromatography-tandem mass spectrometry (LC–MS/MS) analysis.

The metabolites were analysed by ultra-performance liquid chromatography (UPLC) (Shim-pack UFLC SHIMADZU CBM30A, http://www.shimadzu.com.cn/) and MS/MS (AB SCIEX 6500 QTRAP) under the following conditions described by Li and Song^46^: column, water ACQUITY UPLC HSS T3 C18 1.8 μm, 2.1 × 100 mm; mobile phase, the aqueous phase was ultrapure water (0.04% acetic acid), and the organic phase was acetonitrile (0.04% acetic acid); water/acetonitrile gradient, 95:5 V/V for 0 min, 5:95 V/V for 11.0 min, 5:95 V/V for 12.0 min, 95:5 V/V for 12.1 min, and 95:5 V/V for 15.0 min; flow rate, 0.4 mL/min; column temperature, 40 °C; and injection volume, 2 μL. The electrospray ionization (ESI) temperature was 500 °C, the MS voltage was 5,500 V, the curtain gas (CUR) was 25 psi, and the collision-induced dissociation (CAD) parameter was set to high. In triple quadrupole mode (QQQ), each ion pair was scanned for detection based on the optimized decompression potential (DP) and collision energy (CE)^47^.

### Qualitative and quantitative determination of metabolites

Qualitative and quantitative MS analysis of the metabolites in the samples was performed using the self-built MetWare database (MWDB) (MetWare Company, Wuhan, China) and multiple reaction monitoring (MRM)^47^. The material was qualitatively analysed based on secondary spectral information, and the isotope and repetitive signals were removed during the analysis. The metabolites were quantified using MRM with QQQ MS. The ions corresponding to substances with different molecular weights were excluded, and the precursor ions of the target substance were screened. In addition, in the collision cell, the precursor ions were ionized to break and form fragment ions, and the characteristic fragment ions were selected by QQQ filtration. These steps increase the accuracy and repeatability of the quantitative results. Peak area integration was performed for the obtained mass spectral peaks of the metabolites, and the mass spectral peaks of the metabolites from different samples were integrated^48^.

### Scanning electron microscopy

Scanning electron microscopy was performed according to a previously published protocol^43^. The leaves and corollas were cut into 3–5-mm^2^ pieces, fixed in 4% glutaraldehyde in 0.1 mol/L PBS (pH 7.2) for 4 h at 4 °C, washed three times with the same buffer, postfixed in 1% osmium tetroxide for 2 h at room temperature and rinsed three times with the same buffer. The samples were dehydrated with an increasing ethanol series and then dried with a critical point dryer (CPD 030, Switzerland, Bal-Tec). The dried samples were fixed on the sample stage, coated with gold using ion sputtering equipment, observed with a scanning electron microscope (XL-30-ESEM, The Netherlands, FEI) at an acceleration of 10 kV and photographed.

### Transmission electron microscopy

The corolla limbs and leaves from *PhCS*-silenced and control plants were cut into < 1-mm^2^ pieces. The samples were fixed, dehydrated, cut and stained according to a previously published protocol^43^. The samples were visualized with a Tecnai 12 transmission electron microscope (FEI, Eindhoven, The Netherlands) at an acceleration of 80 kV and photographed.

### Folate measurement

Measurement of the folate content was essentially performed as described previously (Navarrete et al. 2012), with some modifications. Briefly, 50-mg (fresh weight) amounts of petunia leaves, sepals and corollas were separately extracted, including deconjugation, for folate analysis, and the resulting extracts were subjected to separation using UPLC (for folates) followed by MS/MS using ESI. The following standard concentrations were used: 0 nmol/L, 1.5 nmol/L, 3 nmol/L, 6 nmol/L, 12 nmol/L, and 24 nmol/L. The final quantitative data for folates reflect the sum of six different folate monoglutamates: 5-methyltetrahydrofolate (5-MTHF), 10-formylfolic acid (10-CHOFA), 5,10-methenyltetrahydrofolate (5,10-CH + THF), folic acid (FA), tetrahydrofolate (THF) and 5-formyltetrahydrofolate (5-CHOTHF).

### Statistical analyses

The statistical analyses of the data were performed using one-way analysis of variance (ANOVA) followed by Duncan’s multiple range test (DMRT) with at least three replicates. *P* values ≤ 0.05 were considered to indicate significance.

## Results

### Isolation and sequence analyses of *PhCS*

To acquire the petunia *CS* sequence, we performed BLAST searches of the *Petunia axillaris* draft genome sequence v1.6.2 (https://solgenomics.net/organism/Petunia_axillaris/genome) using the coding sequence of *A. thaliana AtCS* (At1g48850) as the query, and only one petunia *CS* homologue, named *PhCS*, was recovered. The full-length *PhCS* coding sequence, which was predicted to encode a 439-amino-acid protein with a calculated molecular weight of 47.4 kDa, was isolated from petunia ‘Ultra’. The multiple sequence alignments of CSs of petunia, *A. thaliana*, *Solanum tuberosum*, *Solanum lycopersicum*, *Nicotiana tabacum*, *Pisum sativum*, *Corydalis sempervirens*, *Oryza sativa* and *Escherichia coli* are shown in Fig. S1. Phylogenetic trees based on evolutionary distances were constructed from the CS amino acid sequences using the DNAMAN program (Fig. S1). The deduced amino acid sequences of PhCS exhibited 93.2%, 94.3%, 89.3%, 94.7%, 90.8%, 79.0%, 76.7%, 77.4%, 70.7%, and 48.8% identity with NtCS (accession number: NP_001312420.1), SlCS1 (NP_001234422.1), SlCS2 (NP_001234411.1), StCS1 (XP_006340574.1), StCS2 (XP_006359349.1), AtCS (NP_564534.1), CsCS (CAA43034.1), PsCS (Psat6g184400.1, https://urgi.versailles.inra.fr/Species/Pisum), OsCS2 (XP_015633078.1) and EcCS (WP_137595377.1), respectively (Fig. S2).

The N-terminal regions of the CS showed low similarity but indicated the presence of plastidic transit peptides in higher plants^27^. PhCS contained a high proportion of hydroxylated amino acids (13 out of 50), which is a common feature of plastidic transit peptides^49^. The amino acid I^50^QAA^53^ in the PhCS showed similarity with the consensus sequence (V/I)X(A/C)A for cleavage sites (I) of plastidic transit peptides^50^. The structure of the predicted PhCS contained a conserved FMN-binding site (cd07304) and the highly conserved regions CS1 (PS00787), CS2 (PS00788), and CS3 (PS00789) (Fig. S1). We also found one or two tripeptide (SRL and SKL) peroxisomal targeting signals (PTSs) in the plastidic transit peptides of some CS proteins, including PhCS, SlCS1, StCS1 and NtCS1 but not SlCS2, StCS2, AtCS and PsCS (Fig. S1).

### PhCS protein localization in chloroplasts and peroxisomes

**Figure 2:**
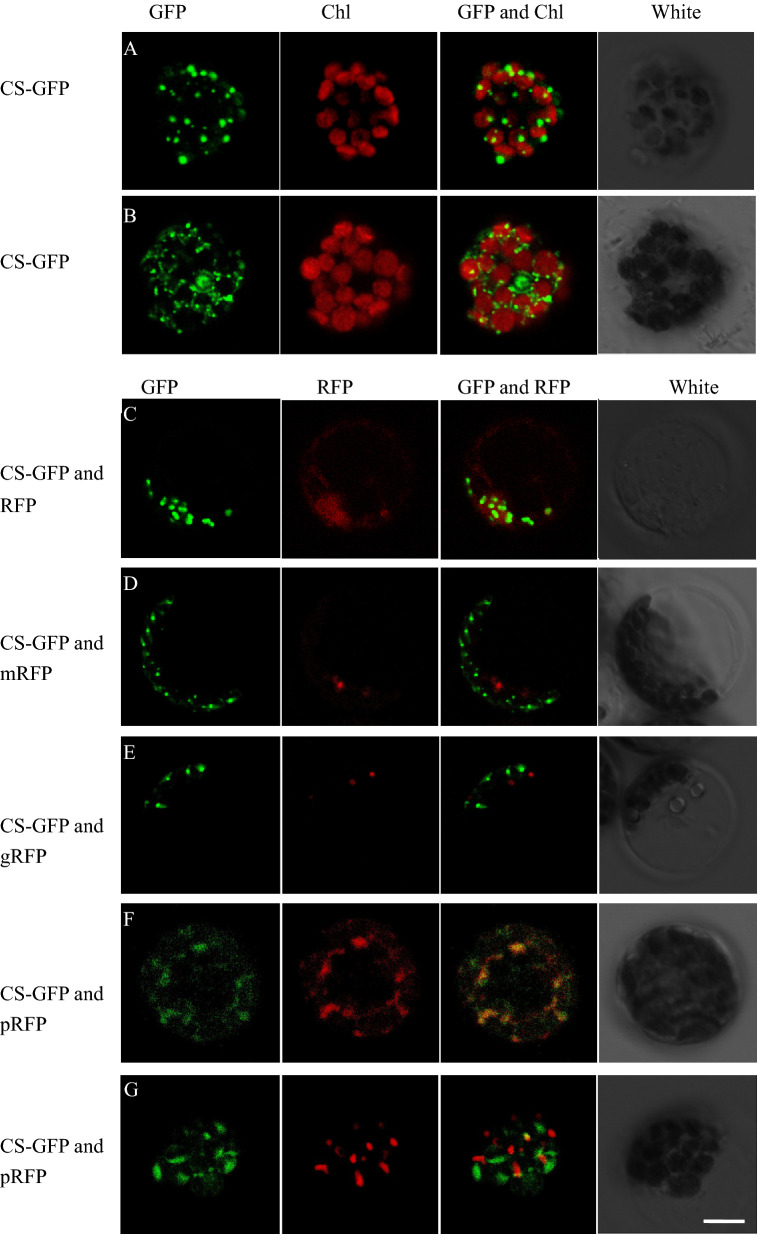
Subcellular locations of PhCS. PhCS C-terminal GFP fusion proteins were transiently expressed in petunia protoplasts coexpressing gRFP, mRFP and pRFP, which served as Golgi, mitochondrion and peroxisome markers, respectively, and visualized by confocal microscopy. (**A**,**B**) CS-GFP chloroplasts were visualized by autofluorescence. Chl, chlorophyll. (**C**) CS-GFP and RFP, (**D**) CS-GFP and mRFP, (**E**) CS-GFP and gRFP, and (**F**,**G**) CS-GFP and pRFP. Scale bars: 5 µm. Images were processed by Zen 2010 (version 6.0, Carl Zeiss Microscopy GmbH, Germany) software.

To examine the subcellular localization of PhCS in plant cells, GFP was fused to the C-terminal end of the full-length PhCS protein to form a PhCS-GFP vector, and the vector was transiently expressed in petunia leaf protoplasts. After 16–24 h, fluorescent signals were observed not only in chloroplasts but also in other organelles (Fig. 2A,B). PhCS-GFP was transiently expressed in petunia protoplasts coexpressing empty RFP and three markers, namely, gRFP, mRFP and pRFP, which were the Golgi, mitochondrion and peroxisome markers, respectively, and visualized by confocal microscopy (Fig. 2C–G). The results showed that PhCS-GFP was partially colocalized with the peroxisome marker pRFP (Fig. 2F,G), which showed that PhCS was also localized in peroxisomes.

### *PhCS* expression

**Figure 3:**
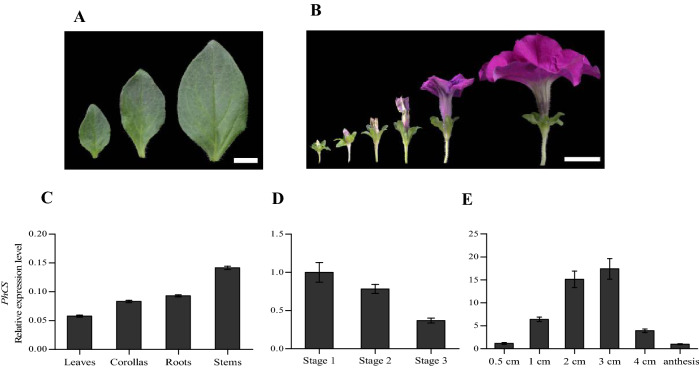
Expression patterns determined by quantitative real-time PCR. (**A**) Three developmental stages of petunia leaves: stage 1 (young leaves, 1.0 cm), stage 2 (growth leaves, 2.5 cm), and stage 3 (mature leaves, 4.0 cm). (**B**) Six stages of petunia flower buds: 0.5 cm, 1 cm, 2 cm, 3 cm, 4 cm and anthesis. (**C**–**E**) Expression of *PhCS* in leaves, corollas, roots, and stems (**C**), in leaves at three stages (**D**) and in corollas during flower development (**E**). (**A**) Bar = 1 cm, (**B**) bar = 2 cm.

The expression of *PhCS* in different plant leaf and flower organs at different developmental stages was examined by quantitative RT-PCR (qPCR) (Fig. 3A,B). A high transcription level of *PhCS* was found in stems, no significant difference in transcription was found between the roots and corollas, and a low expression level was detected in the leaves (Fig. 3C). The expression of *PhCS* gradually decreased during leaf growth (Fig. 3D). During flower development, the expression of *PhCS* increased, peaked when the flower buds were at 3 cm, and then decreased (Fig. 3E).

### VIGS-mediated *PhCS* silencing affects plant development

**Figure 4:**
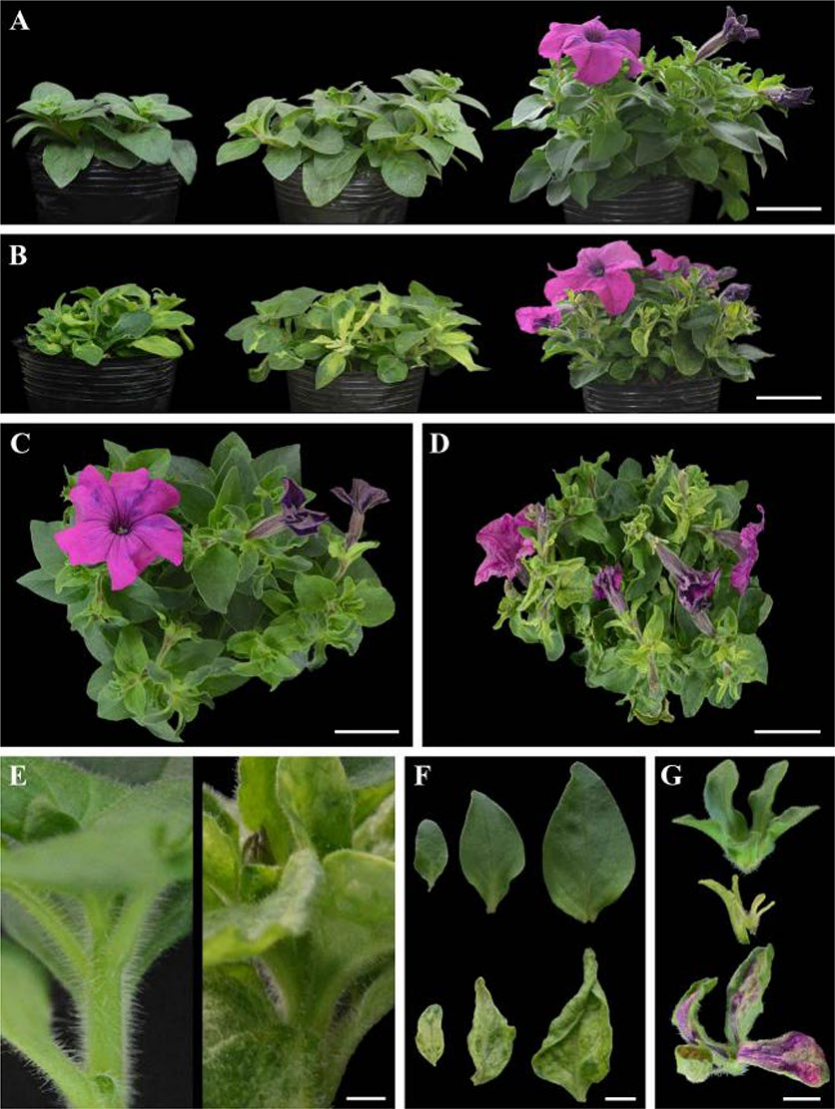
Phenotypic alteration resulting from VIGS of *PhCS* in plants. (**A**,**B**) Two-week-old, 3-week-old and 5-week-old controls are shown from left to right (**A**), and corresponding *PhCS*-silenced plants are shown in (**B**). (**C**,**D**) Five-week-old control plants (**C**) and *PhCS*-silenced plants (**D**). (**E**) Internodes of *PhCS*-silenced (right) and control plants (left). (**F**) Leaves of control (top) and *PhCS*-silenced plants (bottom). (**G**) Mature sepals infected with pTRV2-PhCS (middle and bottom) and control sepals (top). (**A**–**D**) Bar = 5 cm, (**E**) bar = 0.5 cm, (**F**,**G**) bar = 1 cm.

To explore the role of *PhCS* in petunia metabolism, the pTRV2-PhCS vector containing a 291-bp fragment of the 3′ untranslated sequence of *PhCS* cDNA was generated for silencing of *PhCS* by VIGS. One month later, the growth of the pTRV2*-*PhCS-infected plants was stunted (Fig. 4A–D). The stem internode length of the silenced plants was shorter than that of the empty pTRV2-infected control plants, and the mature plants infected with pTRV2-PhCS showed a dwarf phenotype. (Fig. 4E). The leaf blades of the *PhCS*-silenced plants showed upward curling and thickening compared with those of the control plants (Fig. 4F). The colour of the leaves of the *PhCS-*silenced plants was light yellow, whereas that of the control leaves remained green (Fig. 4F). Moreover, *PhCS* silencing affected the sepal colour; specifically, the colour of both the sepals and leaves became light yellow after silencing of *PhCS* (Fig. 4G). qPCR analysis revealed that *PhCS* silencing led to a sharp decline in the expression of *PhCS* in leaves and sepals (Fig. S3A,B).

Because most leaves infected with pTRV2-PhCS turned light yellow, we examined the levels of chlorophyll (Fig. S4A) and carotenoids (Fig. S4B) in these leaves. The levels of both chlorophyll a and b and carotenoids in the *PhCS-*silenced leaves were reduced to only 48.8%, 49.9% and 39.8% of the control levels, respectively (Fig. S4A,B).

### Enzymatic properties of PhCS in pTRV2-PhCS- and pTRV2-treated leaves

**Figure 5:**
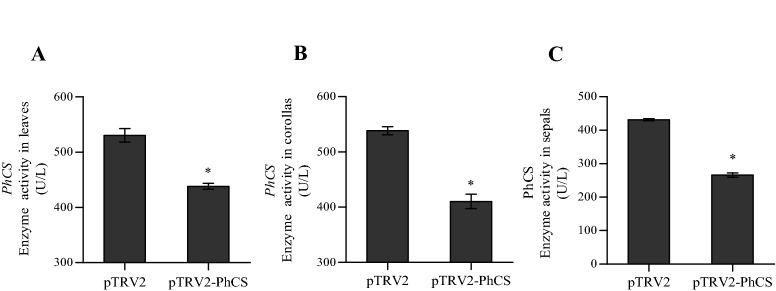
Enzymatic activity of CS in protein extracts. (**A**) Leaves of *PhCS*-silenced and control plants. (**B**) Corollas of *PhCS*-silenced and control plants. (**C**) Sepals of *PhCS*-silenced and control plants. The data are presented as the means ± SDs (n = 3). The statistical analysis was performed using one-way analysis of variance (ANOVA) followed by Duncan’s multiple range test (DMRT) with three biological replicates. *P* values ≤ 0.05 were considered significant.

The enzymatic properties of PhCS in mature leaves, sepals and corollas treated with pTRV2-PhCS and pTRV2 were determined using the Chorismate Synthase Activity Assay Kit with the proteins. The results showed that *PhCS* silencing significantly reduced the activities of CS in the leaf, sepal and corolla protein extracts compared with the control (Fig. 5A–C).

### Effects of *PhCS* silencing on flower organs

**Figure 6:**
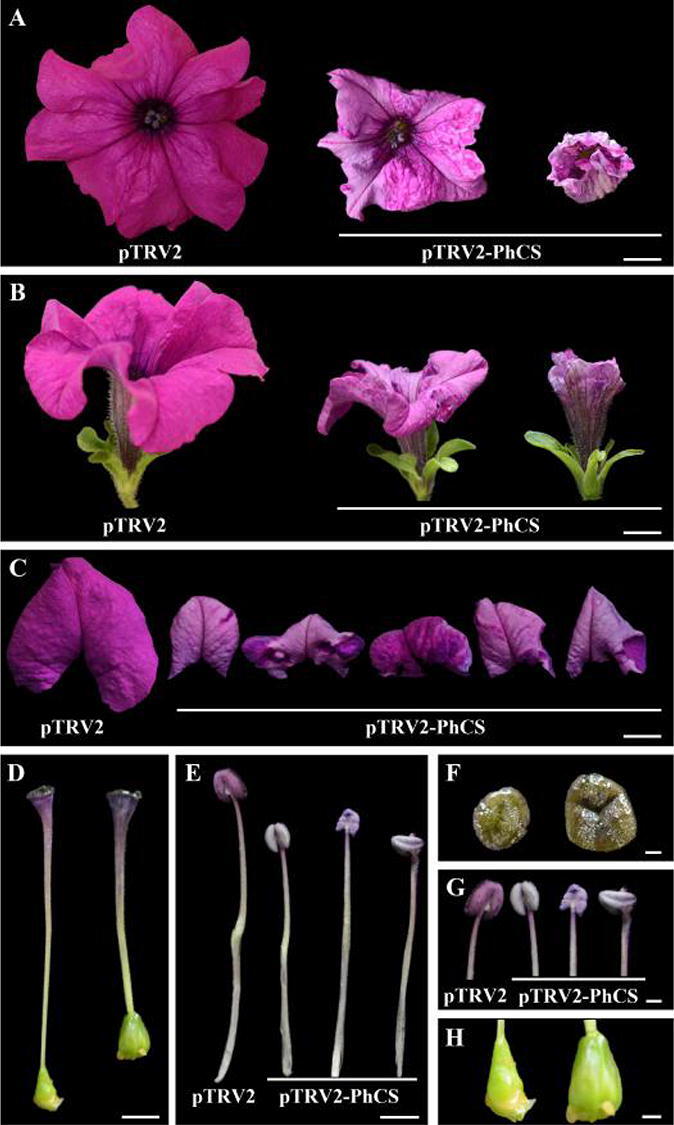
Effects of *PhCS* silencing on flowers. (A, B) Top views (**A**) and side views (**B**) of *PhCS*-silenced opened flowers (middle and right) and control flowers (left). (**C**) Lobes of control and *PhCS*-silenced corollas. (**D**) Pistils of controls (left) and *PhCS*-silenced plants (right). (**E**,**F**) Stamens of control and *PhCS*-silenced plants (**E**) and a few anthers of *PhCS*-silenced plants (lower) and corolla tubes joined together (**F**). (**G**–**I**) Ovary (unpollinated) (**G**), stigma (**H**), and anthers (**I**) of *PhCS*-silenced (right) and control plants (left). (**A**–**C**) Bar = 2 cm, (**D**,**E**) bar = 0.2 cm, (**F**) bar = 1 mm, (**G**) bar = 0.1 cm, (**H**) bar = 3 mm.

Four weeks after infection, the flowers of the pTRV2-PhCS-infected plants exhibited abnormal development and light-coloured corollas, although plants with reduced CS activity did not show a change in the flowering time or total number of flowers (Fig. 4C,D). The reduced CS activity exerted a severe effect on flower development: the size of the flowers was small, some flower buds remained small, and some flower buds did not undergo further development. The sizes of both the corollas and sepals were significantly reduced by *PhCS* silencing (Fig. 6A,B). The diameter of the corollas of the pTRV2-PhCS-infected plants was 73.0% that of the pTRV2-infected corollas (Table 1). The margins of the corollas of the *PhCS*-silenced plants curled downward (Fig. 6C). In addition, the filaments and styles of the *PhCS*-silenced plants were shorter than those of the control plants (Fig. 6D,E); specifically, the lengths of the styles and filaments of the *PhCS*-silenced plants were 78.1% and 46.0% those of the control plants, respectively (Fig. 6D,E). Moreover, after *PhCS* silencing, the diameter of the stigmas increased, some anthers became deformed, and the unpollinated ovaries increased in size (Fig. 6F–H).

**Table 1:** Effects of *PhCS* silencing on petunia plant growth.

	Wild type	PhCS silencing	PhCS/CK (%)
Height of plant (cm)	13.58 ± 0.58	10.58 ± 0.55*	77.9
Diameter of plant (cm)	29.51 ± 0.55	21.44 ± 1.25*	72.7
Length of internode (cm)	1.74 ± 0.1	0.77 ± 0.06*	44.3
Length of mature leaf (cm)	4.99 ± 0.18	4.06 ± 0.24 *	81.4
Width of mature leaf (cm)	3.04 ± 0.07	2.08 ± 0.19*	68.4
Length of pedicel (cm)	3.44 ± 0.13	2.45 ± 0.27*	71.2
Diameter of corolla (cm)	6.78 ± 0.14	4.95 ± 0.28*	73.0
Length of filament (cm)	2.02 ± 0.09	1.63 ± 0.06*	46.0
Length of style (cm)	2.19 ± 0.06	1.71 ± 0.03*	78.1
Diameter of stigma (mm)	2.01 ± 0.03	2.75 ± 0.05*	136.8
Width of ovary (mm)	3.52 ± 0.02	4.49 ± 0.08*	127.6

*Data are the means ± SEs from 15 to 20 samples. Statistical analysis was performed using Student’s *t* test with 15 to 20 replicates. Asterisks indicate significant differences at the P ≤ 0.05 level.

**Figure 7:**
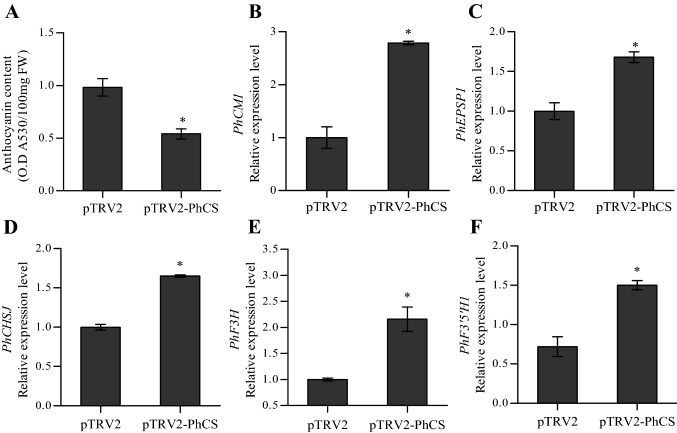
Effects of pTRV2-PhCS treatment on the anthocyanin content and the expression of *PhEPSPS1*, *PhCM1*, *PhCHSJ*, *PhF3H* and *PhF3′5′H1* in corollas. *Cyclophilin* (accession no. EST883944) was used as the internal reference gene for quantification of cDNA abundance. The data are presented as the means ± SDs (n = 3). The statistical analysis was performed using one-way analysis of variance (ANOVA) followed by Duncan’s multiple range test (DMRT) with three biological replicates. *P* values ≤ 0.05 were considered significant.

We examined the anthocyanin content in the plant corollas and found that the anthocyanin content of the pTRV2*-*PhCS-infected corollas was 55.1% that of the control corollas (Fig. 7A). In addition, the *PhCS* mRNA level in the soft pink corollas of the *PhCS*-silenced plants was significantly reduced compared with the control levels (< 20%), which indicated that pTRV2-PhCS treatment decreased the *PhCS* mRNA levels in corollas (Fig. S3C). *PhCS* silencing also affected the colour of the stamens (Fig. 6G); specifically, silencing of *PhCS* changed the colour of both the stamens and the sepals to a lighter colour (Fig. 6G).

We further analysed the effects of *PhCS* silencing on the transcript levels of *PhEPSPS1*, *PhCM1*, *PhCHSJ, PhF3H* and *PhF3′5′H1* in corollas, and qPCR analysis revealed that *PhCS* silencing induced significant increases in the *PhEPSPS1*, *PhCM1*, *PhCHSJ* and *PhF3H1* mRNA levels (Fig. 7B–F).

### Changes in the corolla metabolome induced by *PhCS* silencing

**Figure 8:**
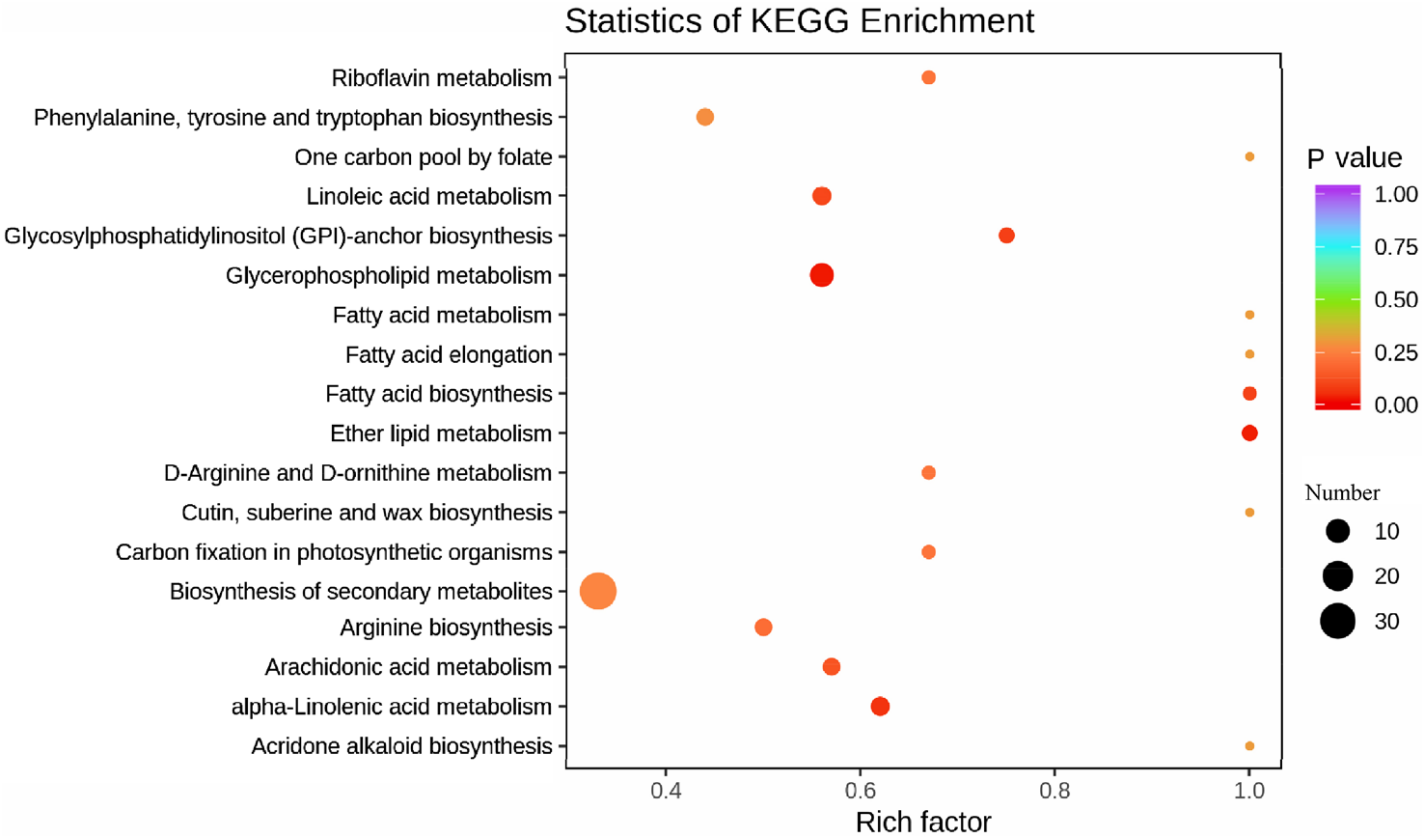
KEGG enrichment analysis of the differentially abundant metabolites in *PhCS*-silenced and control petunia corollas. Data were processed by ggplot2 (version 3.3.0, https://CRAN.R-project.org/package=ggplot2) software.

To further analyse the effect of *PhCS* silencing on the anthocyanin content, widely targeted metabolites in corollas from *PhCS*-silenced and control plants were detected by UPLC and tandem MS to ensure that the samples were collected from the *PhCS*-silenced corollas, the lighter part of the corollas of the plants treated with pTRV2-PhCS was obtained, and the silencing of *PhCS* in the collected samples was confirmed by a qPCR assay. Qualitative and quantitative MS analysis of the metabolites in the samples was performed based on the KEGG database (https://www.genome.jp/kegg/pathway.html), MWDB database (Metware Biotechnology Co, Wuhan, China), and MRM, and 511 metabolites, including 82 flavonoids, were identified (Fig. 8; Fig. S5A,B, Supplemental Data File 1).

The differentially abundant metabolites were screened, and the criteria used for this screening included a fold change value ≥ 2 or ≤ 0.5 and a VIP value ≥ 1. Thirty-nine and 126 metabolites were downregulated and upregulated, respectively, with a high degree of repeatability (Fig. S6A,B; Supplemental Data File 2). All differentially abundant metabolites were categorized based on the KEGG database. The differentially abundant metabolites in *PhCS*-silenced corollas were mainly enriched in glycerophospholipid metabolism, ether lipid metabolism, alpha-linolenic acid metabolism, glycosylphosphatidylinositol (GPI)-anchor biosynthesis, fatty acid biosynthesis, linoleic acid metabolism, arachidonic acid metabolism, arginine biosynthesis, and carbon fixation in photosynthetic organisms (Fig. 8; Fig. S6A; Supplemental Data File 3).

Among the differentially abundant metabolites, 27 flavonoid metabolites were significantly changed, and 21 of these flavonoids were significantly downregulated. In addition, three and three anthocyanins were downregulated and upregulated, respectively (Supplemental Data File 4). These results are not inconsistent with the reduction in the total anthocyanin content detected in corollas because the levels of different anthocyanin metabolites in corollas exhibit differences^51^.

It is worth noting that *PhCS* silencing did not significantly change the levels of free phenylalanine, tryptophan, and tyrosine in petunia corollas (Supplemental Data File 1). It might be necessary to maintain stable levels of free phenylalanine, tryptophan, and tyrosine in corollas, and the levels of downstream metabolites, such as the total anthocyanin levels and the levels of most flavonoid metabolites, were reduced in *PhCS*-silenced plants compared with the control plants.

In addition, most primary and secondary metabolites in corollas treated with pTRV2-PhCS were significantly upregulated. All 23 differentially abundant amino acids and derivatives (Supplemental Data File 5), all 14 differentially abundant nucleotides and derivatives (Supplemental Data File 6), all six differentially abundant lignans and coumarins (Supplemental Data File 7), all 13 differentially abundant alkaloids (Supplemental Data File 8), all 29 differentially abundant lipids (Supplemental Data File 9), and most (10/11) organic acids (Supplemental Data File 10) were significantly upregulated in *PhCS*-silenced plants compared with the control plants. Moreover, half (13/26) of the differentially abundant phenolic acids were upregulated in the *PhCS*-silenced plants compared with the control plants (Supplemental Data File 11).

### *PhCS* silencing impedes cellular expansion

**Figure 9:**
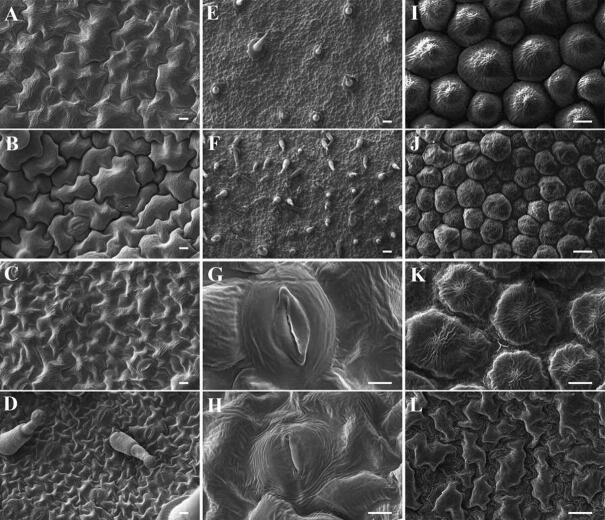
Scanning electron micrographs of the *PhCS*-silenced and control plants. (**A**,**B**) Adaxial leaf epidermal cells of control (**A**) and *PhCS*-silenced plants (**B**). Bars = 10 μm. (**C**,**D**) Abaxial leaf epidermal cells of control (**C**) and *PhCS*-silenced plants (**D**). Bars = 10 μm. (**E**,**F**) Trichomes of abaxial leaf epidermal cells in control (**E**) and *PhCS*-silenced plants (**F**). Bar = 100 μm. (**G**,**H**) Stoma of abaxial leaf epidermal cells in control (**G**) and *PhCS*-silenced plants (**H**). Bar = 2 μm. (**I**,**J**) Adaxial corolla epidermal cells of control (**I**) and *PhCS*-silenced plants (**J**). Bars = 10 μm. (**K**,**L**) Abaxial corolla epidermal cells of control (K) and *PhCS*-silenced plants (**L**). Bars = 10 μm.

To further characterize the role of *PhCS*, the surface ultrastructures of plants treated with pTRV2 and pTRV2-PhCS were characterized. As observed by scanning electron microscopy, the adaxial leaf epidermal cells of the treated plants showed an obviously reduced cell length and a slightly reduced cell width (Fig. 9A,B), and the length and width of abaxial leaf epidermal cells were also decreased (Fig. 9C,D). Moreover, we found that the density of trichomes on the abaxial surfaces of pTRV2-PhCS-infected leaves was notably higher than that of trichomes on the abaxial surfaces of pTRV2-infected leaves (Fig. 9E,F). Silencing of *PhCS* also reduced the size of the stomata of abaxial epidermal cells but did not exert a significant effect on the size of the stomata of adaxial epidermal cells (Table 2). The stomata of abaxial epidermal cells infected with pTRV2-PhCS were smaller than those of cells infected with pTRV2, and in particular, the results showed that the former exhibited a decreased width (Fig. 9G,H). The sizes of the adaxial and abaxial epidermal cells of the corollas of *PhCS*-silenced plants were reduced, and these cells were deformed (Fig. 9I–L).

**Table 2:** Effects of *PhCS* silencing on petunia cells.

	pTRV2	pTRV2-PhCS	pTRV2-PhCS/pTRV2 (%)
Length of adaxial epidermal cells of leaves (µm)	78.97 ± 4.52	53.43 ± 2.00*	67.7
Width of adaxial epidermal cells of leaves (µm)	50.89 ± 0.83	38.98 ± 2.75*	76.6
Length of abaxial epidermal cells of leaves (µm)	65.67 ± 2.70	20.84 ± 1.88*	31.7
Width of abaxial epidermal cells of leaves (µm)	56.15 ± 1.47	14.33 ± 1.02*	25.2
Diameter of adaxial epidermal cells of petal limbs (µm)	24.01 ± 0.75	16.78 ± 0.74*	69.9
Height of adaxial epidermal cells of petal limbs (µm)	31.93 ± 0.85	14.35 ± 1.93*	44.9
Diameter of abaxial epidermal cells of petal limbs (µm)	23.89 ± 0.86	16.31 ± 0.68*	68.3
Height of abaxial epidermal cells of petal limbs (µm)	15.87 ± 2.06	16.78 ± 0.62	105.7
Length of adaxial epidermal cells of stomata (µm)	19.88 ± 0.52	21.03 ± 0.60	105.8
Width of adaxial epidermal cells of stomata (µm)	16.12 ± 0.60	16.15 ± 0.36	100.2
Length of abaxial epidermal cells of stomata (µm)	20.73 ± 0.79	18.21 ± 0.95	87.8
Width of abaxial epidermal cells of stomata (µm)	17.35 ± 0.69	12.27 ± 0.60*	70.7

The mean values ± SEs of three biological replicates are shown. *P ≤ 0.05 as determined by Student’s *t* test of *PhCS*-silenced plants compared with the control.

In addition, transmission electron micrographs showed that *PhCS* silencing changed the shape and alignment of the adaxial and abaxial epidermal cells of corollas compared with those of the control plants (Fig. S7). Detailed observations displayed sharp protrusions from the adaxial epidermal cells of the pTRV2-infected plants, whereas the adaxial epidermal cells of the pTRV2-PhCS-infected corollas exhibited smooth surfaces (Fig. S7A). In addition, the size of the adaxial epidermal cells was smaller than that of the controls.

### *PhCS* silencing decreases the total folate content in leaves

Chorismate is a central metabolite in plant cells that serves as an initiator substrate for the synthesis of tetrahydrofolate^52^. To investigate whether *PhCS* silencing affects the folate biosynthesis pathway, we examined the total folate content in leaves, sepals and corollas. The highest and lowest folate levels were found in leaves and corollas, respectively (Fig. S8). The folate content in the leaves and sepals of *PhCS*-silenced plants was lower than that found in the control plants, whereas only a small difference was found in the corollas, which indicated that *PhCS* might play an important role in folate biosynthesis in leaves and sepals (Fig. S8).

### MTX treatment reduces the levels of chlorophyll and folate in leaves

MTX is a dihydrofolate analogue that specifically inhibits THF synthesis, and the target of MTX is dihydrofolate reductase. In this study, we sprayed MTX (100 mM) on the surface of the leaves and analysed the resulting effects on the levels of chlorophyll and total folate. MTX treatment resulted in yellow leaves and sepals (Fig. S9A–C). Chlorophyll and folates were extracted and analysed^54^, and the results showed that the total folate and chlorophyll levels in the leaves were significantly reduced (Fig. S10A,B).

## Discussion

CS has been extensively studied in prokaryotes and fungi, but relatively little is known regarding CS in higher plants. The enzymatic reaction catalysed by CS is the last step in the shikimate pathway^28^, and most petunia corollas synthesize large quantities of anthocyanins, which are derived from phenylalanine^55^. In the present study, we analysed the localization of PhCS and obtained a *PhCS*-silenced petunia phenotype using VIGS, and our results demonstrated that *CS* plays important roles in plant growth and development.

Full-length cDNAs for *CSs* have been isolated from a number of organisms, including *Arabidopsis*, *Corydalis sempervirens*, tomato, *Euglena gracilis*, and *Mycobacterium tuberculosis*^27,56–58^. Studies have indicated that CSs are encoded by a single gene or a small gene family^28^. The amino acid identities between the petunia PhCS enzyme and the *A. thaliana* and *O. sativa* enzymes were found to be 79.0% and 70.7%, respectively, which suggests that CS is highly conserved in higher plants. The structures of these predicted CSs contain conserved FMN-binding sites (cd07304) and the highly conserved CS1 (PS00787), CS2 (PS00788), and CS3 (PS00789) regions, which indicates that these enzymes have similar catalytic functions (Fig. S1). In addition, one or two tripeptide (SRL and SKL) PTSs are found in the plastidic transit peptides of some CS proteins, including PhCS.

The CSs of most higher plant species have plastidic transit peptides, which indicates that CS is localized in chloroplasts. Moreover, PsCS activity in pea has been detected only in chloroplasts and not in cytoplasmic extracts^6^, which indicates that PsCS is specifically localized in chloroplasts. Notably, no PTS has been found in PsCS (Fig. S1). In this study, we found that PhCS has both a plastidic transit peptide and two PTSs. GFP fusion protein assays have shown that PhCS is localized in both chloroplasts and peroxisomes.

Based on sequence analysis, CS proteins of only some species have one or two PTSs, and even some single-copy CSs in some species, such as *Arabidopsis*, do not have PTSs, which indicates that peroxisome localization of CS might not be necessary for plant growth and development.

PhCM and PhEPSPS cDNAs have been isolated from petunia. PhCM1 and PhEPSPS1 are plastid-localized proteins, as demonstrated by the presence of the cTP sequence (ChloroP 1.1) and a chloroplast import assay. PhCM2 is not localized in chloroplasts^19^, and PhCM2 does not contain a PTS. Two PhEPSPSs (Peaxi162Scf00959g00022 and Peaxi162Scf00013g03114) do not have a PTS, and one PhEPSPS is specifically localized in chloroplasts^13^. These findings indicate that peroxisome-localized PhCS is separated from the substrate and other pathway proteins under normal growth conditions. The specific function of peroxisome-localized CSs needs further study.

In tomato, the two genes are predominantly expressed in the flowers and roots and, to a lesser extent, in stems, leaves, and cotyledons. In petunia, the expression of *PhCS* changes both spatially and temporally during plant development. In addition, during flower development, the expression of *PhCS* increased, peaked when the flower buds were 3 cm and then decreased (Fig. 3E), which supported the role of PhCS in stem elongation and anthocyanin synthesis.

Previous studies have demonstrated that disruption of plastidial shikimate biosynthesis in potato plants by antisense inhibition of DAHP synthase leads to a stunted growth phenotype characterized by reductions in the length, width, and lignin content of the stems^60^. The RNAi-mediated reduction in the expression of NtDHD/SHD-1 in tobacco also leads to a stunted phenotype. In the present study, pTRV2-PhCS-treated plants exhibited a significant reduction in *PhCS* expression and CS activity compared with pTRV2-treated plants. *PhCS* silencing resulted in a short internode length in stems and abnormal leaves and flowers. Consistent with this, the silencing of *PhCS* reduced the epidermal cell sizes of leaves and corollas. It is possible that the chorismate reduction induced by *PhCS* silencing resulted in a reduction in the content of lignin, which plays important roles in the maintenance of structural integrity^61^, in stem elongation and in the formation of leaves and flowers.

A previous study showed that a 50% reduction in DHD/SHD activity results in strongly bleached leaves^17^. In this study, *PhCS* silencing resulted in a loss in the green colour of the leaves and reduction in the carotenoid and chlorophyll levels. The silencing of *PhCS* decreased the leaf and sepal levels of folates, which play an important role in chlorophyll synthesis. The synthesis of chlorophyll in higher plants requires a methylation step that transforms Mg-protoporphyrin IX to Mg-protoporphyrin IX monomethyl ester and a methyl unit^62,63^. AdoMet is the methyl donor of these methyl units and is synthesized and recycled in the cytosol from methyl-THF (CH_3_-THF). In this study, MTX treatment resulted in yellow leaves and sepals and reduced folate and chlorophyll levels (Fig. S10A,B). These results indicated that the reduction in chlorophyll synthesis detected in *PhCS*-silenced plants was due to the decreased folate content, but the cause of the reduction in the carotenoid content requires further study.

Previous studies have documented the importance of the shikimate pathway in the synthesis of plant secondary compounds, including anthocyanins, indole acetic acid and antimicrobial compounds, which have been demonstrated to play vital roles in plant defence^64–66^, wound healing^67^, and maintenance of both the structural integrity and water transport capacity^61^. The inhibition of DHD/SHD leads to reduced chlorogenate and lignin levels. In this study, most flavonoids and the total anthocyanins were reduced in *PhCS*-silenced flowers (Supplemental Data File 4; Fig. 7A) because chorismate and phenylalanine are the precursors of flavonoid and total anthocyanin synthesis^28^. Therefore, PhCS can be considered an important enzyme in the synthesis of flavonoids and anthocyanins. *PhCS* silencing slightly increased the expression of *PhEPSPS1*, *PhCM1*, *PhCHSJ*, *PhF3′5′H1* and *PhF3H* (Fig. 7B–F), which indicates the existence of feedback regulation of the expression of these genes by anthocyanins or other products of the shikimate pathway.

All differentially abundant lignans/coumarins, alkaloids, and some flavonoids (Supplemental Data Files 4, 7, and 8), which are the end products of the shikimate pathway, were upregulated in *PhCS*-silenced corollas (Fig. S1). Given the centrality and complexity of PhCS-associated metabolism, these pleiotropic effects are not necessarily surprising but might reveal novel metabolic regulation connections. Prior studies have shown that lignans/coumarins^68,69^, alkaloids^70–73^, lipids^74,75^, and flavonoids^76^ hyperaccumulate during exposure to many different stresses, which might indicate that these *PhCS*-silenced plants tolerate physiological stress. Similarly, half (13/26) of the differential phenolic acids, which are also the end products of the shikimate pathway, were upregulated in response to the physiological stress induced by *PhCS* silencing. The perception of this stress might prioritize the commitment of the limited chorismate pool to specific downstream products.

Our metabolomic analysis showed that all differentially abundant lipids (Supplemental Data File 9), including fatty acids, were upregulated in *PhCS*-silenced corollas. It is possible that the reduction in many flavonoids resulted in the accumulation of malonyl coenzyme A, and because malonyl-CoA is the common precursor of fatty acids and flavonoids, the carbon flow towards fatty acid synthesis increased^77^. In addition, the lipid composition could be altered under physiological stress yy^74,75,78^.

In conclusion, we found that PhCS is localized in chloroplasts and, unexpectedly, peroxisomes, although the function of peroxisome-localized PhCS remains unknown. The current experiments might provide direct evidence demonstrating the essential role of a plastidial PhCS in the synthesis of phenylalanine and its secondary metabolite derivatives, including anthocyanin and folate. The *PhCS*-silenced plants were also characterized by a substantial growth reduction and deficiencies in photosynthetic pigments. These phenotypes are largely consistent with the results expected following a severe restriction in shikimate biosynthesis. Therefore, future elucidation of the functional role of peroxisome-localized PhCS is likely to provide important insights into the evolution of both shikimate and intermediary metabolism in plants.

## Supplementary information

Supplementary file1 (XLSX 90 kb)

Supplementary file2 (XLSX 9 kb)

Supplementary file3 (XLSX 10 kb)

Supplementary file4 (XLSX 29 kb)

Supplementary file5 (XLSX 16 kb)

Supplementary file6 (XLSX 10 kb)

Supplementary file7 (XLSX 10 kb)

Supplementary file8 (XLSX 9 kb)

Supplementary file9 (XLSX 9 kb)

Supplementary file10 (XLSX 9 kb)

Supplementary file11 (XLSX 11 kb)

Supplementary file12 (PDF 669 kb)
