# Supplementary material for: Suppression of chorismate synthase, which is localized in chloroplasts and peroxisomes, results in abnormal flower development and anthocyanin reduction in petunia
Source: Sci Rep. 2020 Jul 2;10:10846. doi: 10.1038/s41598-020-67671-6 (PMC7331636; doi:10.1038/s41598-020-67671-6)
Supplement: Supplementary file 12 — Supplementary file12 (PDF 669 kb) [file 41598_2020_67671_MOESM12_ESM.pdf]

Supplementary Tables and Figures

Title: Suppression of chorismate synthase, which is localized in chloroplasts and peroxisomes, results in abnormal flower development and anthocyanin reduction in petunia

Shiwei Zhong[1,2], Zeyu Chen[1], Jinyi Han[1], Huina Zhao[3], Juanxu Liu[1], Yixun Yu[1,2]✉

Address: 1, Guangdong Key Laboratory for Innovative Development and Utilization of Forest Plant Germplasm, College of Forestry and Landscape Architecture, South China Agricultural University, Guangzhou 510642, China

2, Lingnan Guangdong Laboratory of Modern Agriculture, Guangzhou, 510642, China

3, College of Horticulture, South China Agricultural University, Guangzhou 510642, China

Name of corresponding author: Yixun Yu

Supplementary information: 3 tables, 10 figures and 11 supplementary dataset files.

Table S1 Primer sequences of *PhCS* used in the subcellular localization analysis

Gene	Forward primer (5′→3′)	Reverse primer (5′→3′)
PhCS	TCACCATTTACGAACGATAG<br>CCATGGCAATGGCGTCGTCGTTTATA<br>C	AGCTCCTCGCCCTTGCTCACCATG<br>GCAAGGGCAACCTCAGCTGACT

Table S2 Primer sequences of *PhCS* and *PhCYP* used in the quantitative real-time PCR assay

Gene	Forward primer (5′→3′)	Reverse primer (5′→3′)
PhCS	TTCCTTTCTCGCCCTTCA	CAACCAACTCCACCACCA
PhCHS	TGGATGAAATGAGAAAGGCTTC	CCACAATATAAGCAACGCCCA
PhF3'5'H1	TGAATTGGGCTGGATCAGT	AGTTGGGCCTTCCTTGTAT
PhF3H	GCCTTAACCAAGGCATGTGT	TAGCTTGAAGCCCACCAACT
PhCYP	AGGCTCATCATTCCACCGTGT	TCATCTGCGAACTTAGCACCG

Table S3 Primer sequences of *PhCS* used in VIGS

Gene	Forward primer (5′→3′)	Reverse primer (5′→3′)
PhCS	GCTGGATCCTGGTGCTTGTGGAT<br>CAGT	CCGGAATTCCTGCATACCCGTGAGT<br>GA

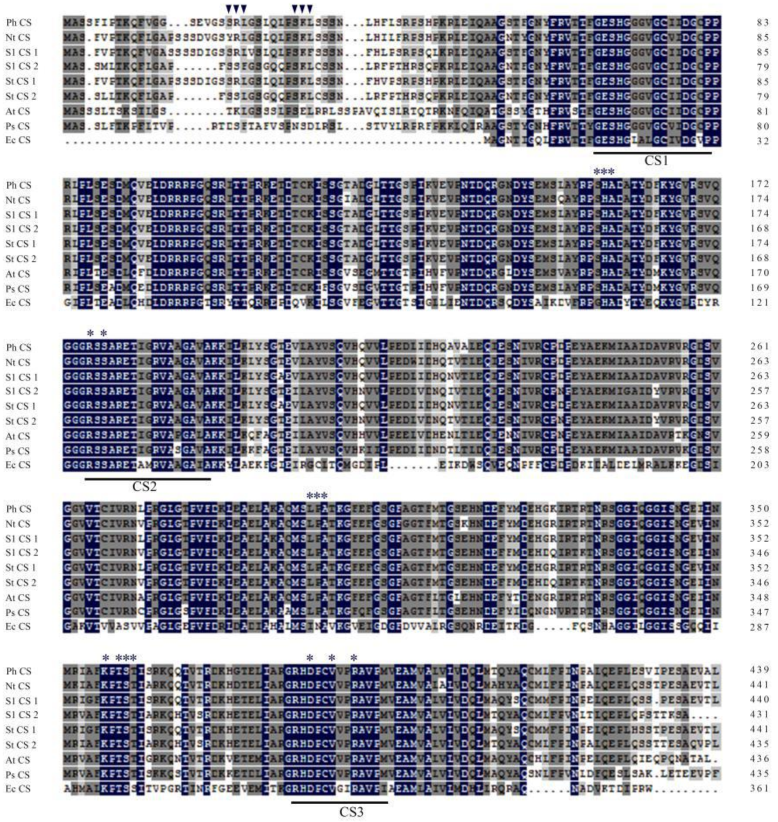

Figure S1. Sequence alignment of CSs. Predicted amino acid sequence alignments of PhCS with *Arabidopsis thaliana* AtCS (AT1G48850), *Solanum tuberosum* StCS1 (XP_006340574.1) and StCS2 (XP_006359349.1), *Solanum lycopersicum* SlCS1 (NP_001234422.1) and SlCS2 (NP_001234411.1), *Nicotiana tabacum* NtCS (NP_001312420.1), *Pisum sativum* PsCS (Psat6g184400.1, https://urgi.versailles.inra.fr/Species/Pisum), and *Escherichia coli* EcCS (WP_137595377.1) using DNAMAN (version 5.2.2, Lynnon Biosoft, America) software. The identical amino acids are shaded in blue, and the conserved changes are shown in grey. The deep grey shading represents identical residues in seven of nine sequences, and the light grey shading indicates five similar residues among nine sequences. The stars above the alignment show the conserved FMN-binding motif, and solid underlines represent the CS1, CS2 and CS3 conserved sites.

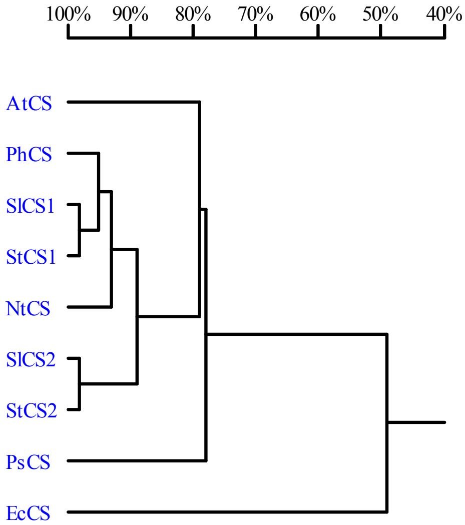

Figure S2. Phylogenetic tree displaying the similarities of CSs among different species. The phylogenetic tree was constructed using DNAMAN (version 5.2.2, Lynnon Biosoft, America) software. The protein names are provided in uppercase letters, and their origin is indicated by a two-letter prefix. The phylogenetic tree of PhCS, *Arabidopsis thaliana* AtCS (AT1G48850), *Solanum tuberosum* StCS1 (XP_006340574.1) and StCS2 (XP_006359349.1), *Solanum lycopersicum* SlCS1 (NP_001234422.1) and SlCS2 (NP_001234411.1), *Nicotiana tabacum* NtCS (NP_001312420.1), and *Pisum sativum* PsCS (Psat6g184400.1) is shown.

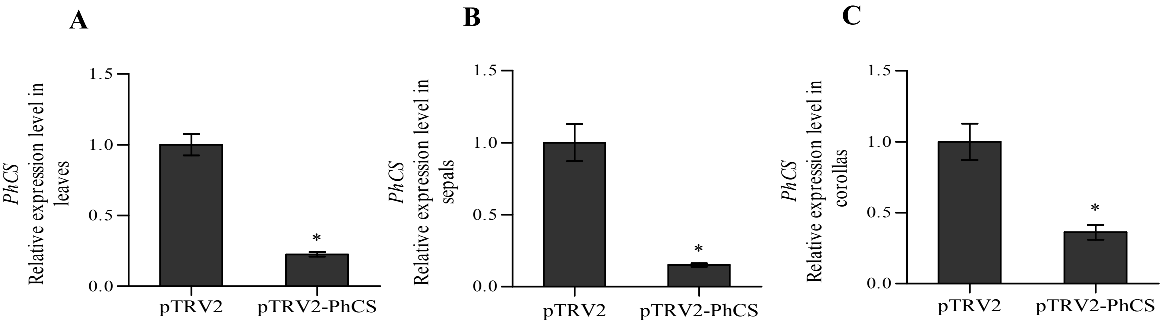

Figure S3. Effects of pTRV2-PhCS treatment on the expression of *PhCS* in leaves, sepals and corollas. *Cyclophilin* (accession no. EST883944) was used as the internal reference gene for quantification of cDNA abundance. The data are presented as the means ± SDs (n = 3). The statistical analysis was performed using one-way analysis of variance (ANOVA) followed by Duncan's multiple range test (DMRT) with three biological replicates. *P*-values ≤ 0.05 were considered significant.

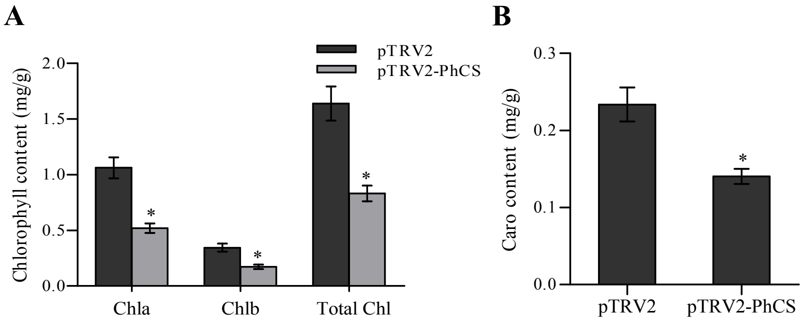

Figure S4. Effects of pTRV2-PhCS treatment on the levels of chlorophylls and carotenoids. Chla, chlorophyll a; Chlb, chlorophyll b; Caro, carotenoid. The data are presented as the means ± SDs (n = 3). The statistical analysis was performed using one-way analysis of variance (ANOVA) followed by Duncan's multiple range test (DMRT) with three biological replicates. *P*-values ≤ 0.05 were considered significant.

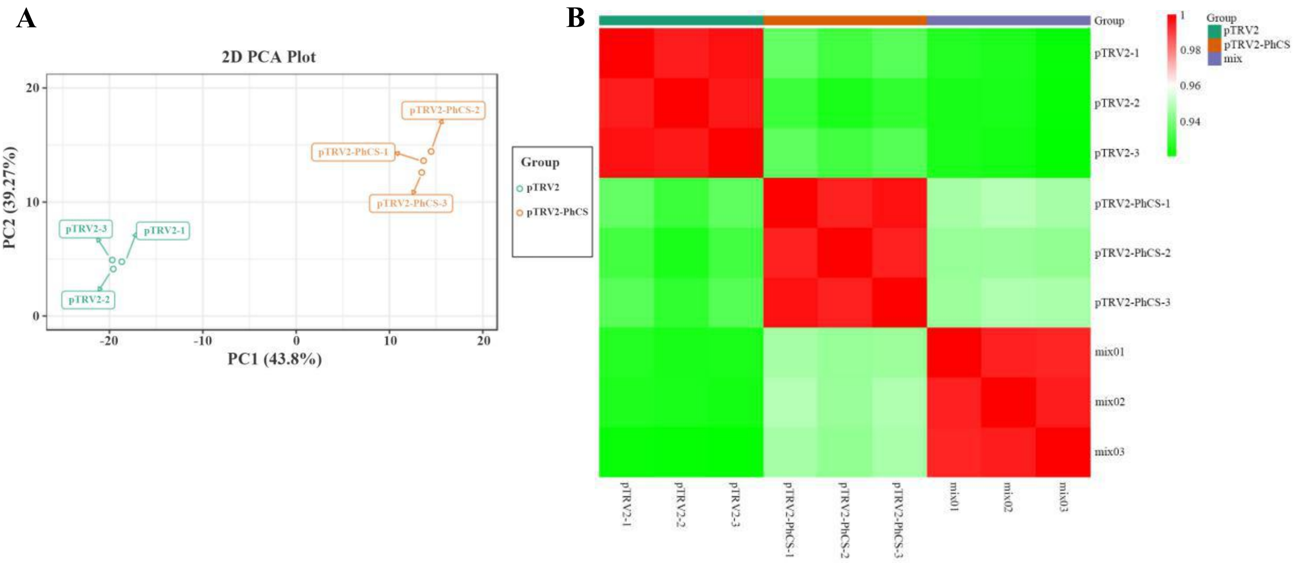

Figure S5. PCA plot and correlation analysis of the metabolome of *PhCS*-silenced and control petunia corollas. A, PCA plot analysis; B, correlation analysis. The images were constructed by R (version 3.5.0, www.r-project.org) software.

A

B

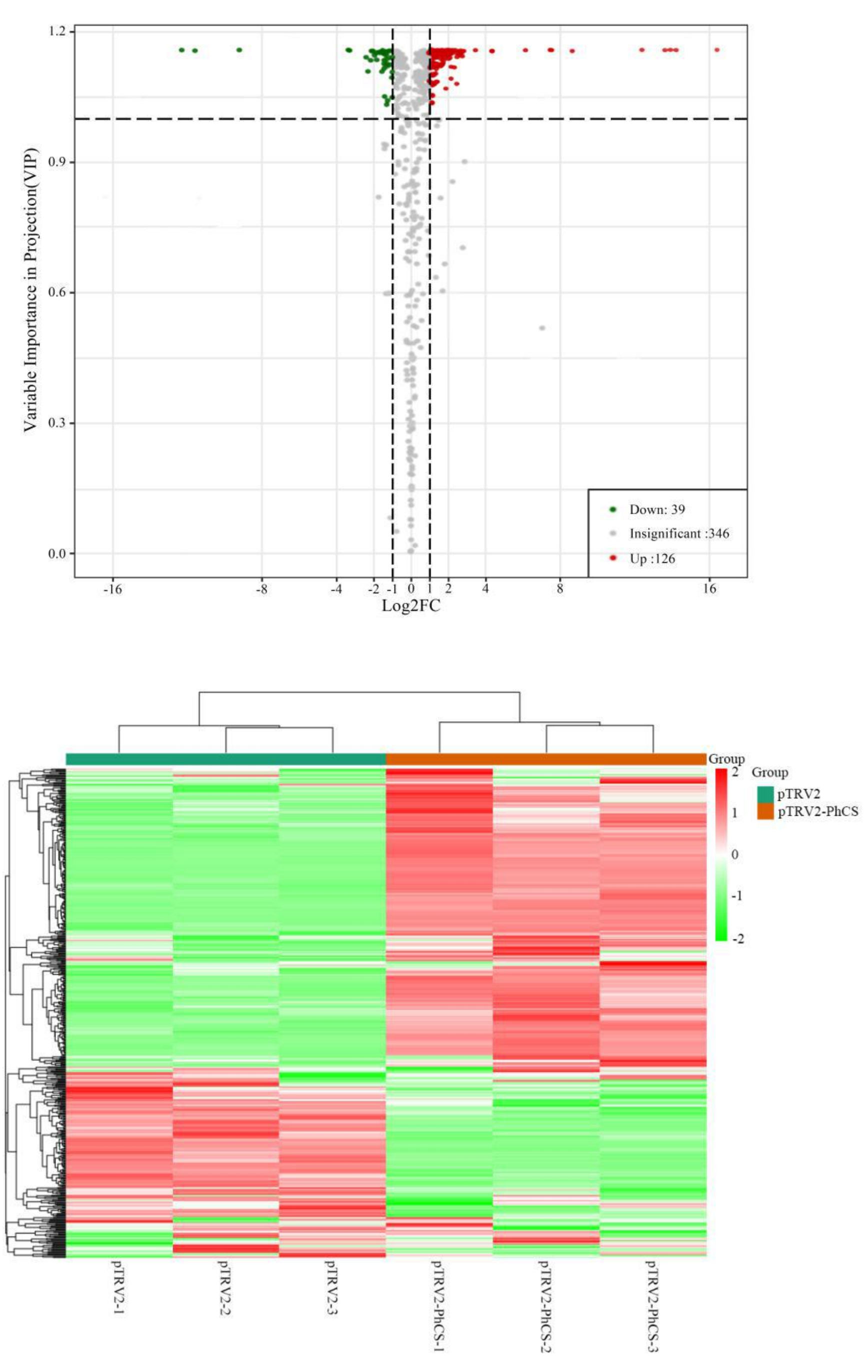

Figure S6. Volcano plot and heat map of the differentially abundant metabolites in *PhCS*-silenced petunia corollas compared with control petunia corollas. A, Volcano plot. The green dots in the figure represent the differentially abundant metabolites that were downregulated, the red dots represent the differentially abundant metabolites that were upregulated, and the black colour indicates metabolites that did not show significant differences. The volcano plot was constructed by the ggplot2 package of R language (version 3.5.0, https://CRAN.R-project.org/package=ggplot2,www.r-project.org) software. B, Heat map. The green colour indicates a decrease in the differentially abundant metabolites, and the red colour

indicates an increase in the differentially abundant metabolites. The image was constructed by pheatmap (version 1.0.12, https://cran.r-project.org/web/packages/pheatmap/) software.

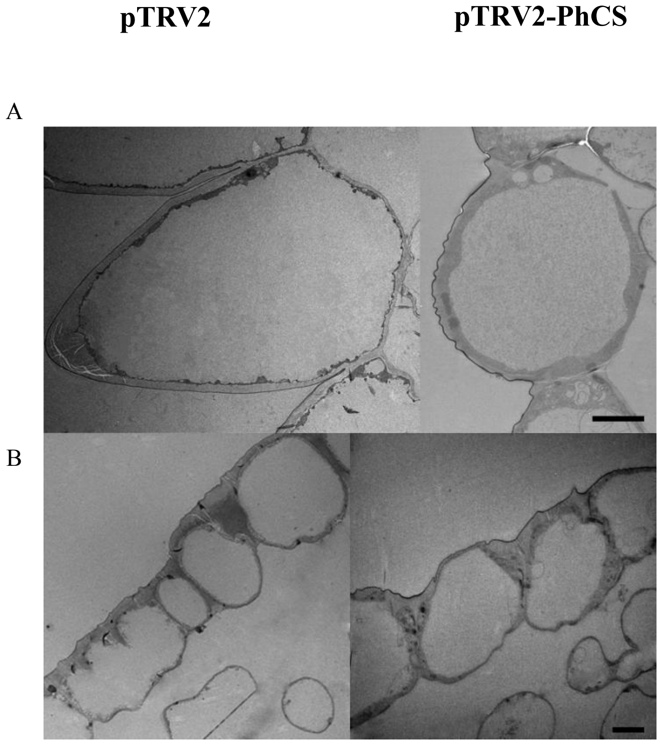

Figure S7. Transmission electron micrographs of the *PhCS*-silenced and control plants. (A) Adaxial corolla epidermal cells of control (left) and *PhCS*-silenced plants (right). (B) Abaxial corolla epidermal cells of control (left) and *PhCS*-silenced plants (right). Bar = 5 μm.

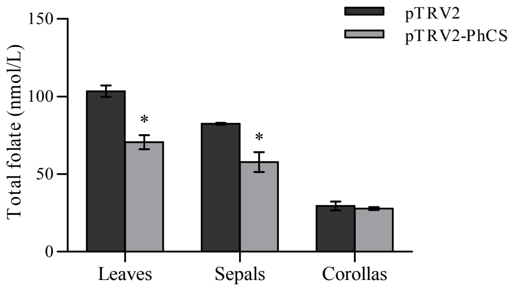

Figure S8. Effects of *PhCS* silencing on the folate content in the leaves, sepals and corollas. The data are presented as the means ± SDs (n = 3). The statistical analysis was performed using one-way analysis of variance (ANOVA) followed by Duncan's multiple range test (DMRT) with three biological replicates. P-values ≤ 0.05 were considered significant.

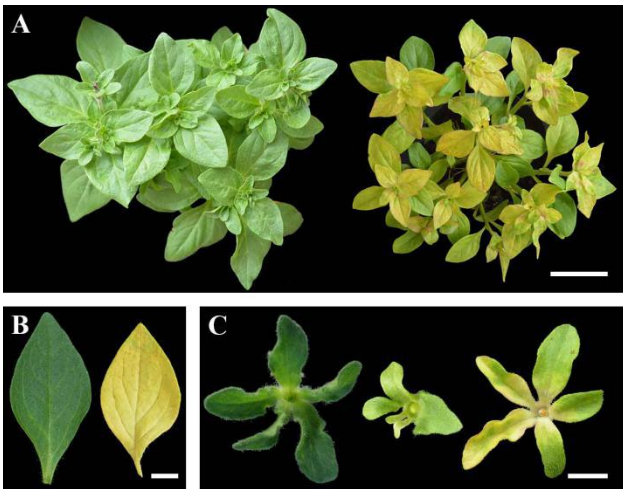

Figure S9. Effects of MTX treatment on the leaves and sepals. (A) Plants treated with MTX (left) and control plants (right). (B) Leaves of control (upper) and MTX-treated (lower) plants. (C) Sepals of control (left) and MTX-treated (middle and right) plants. (A) Bar = 5 cm, (B, C) bar = 1 cm.

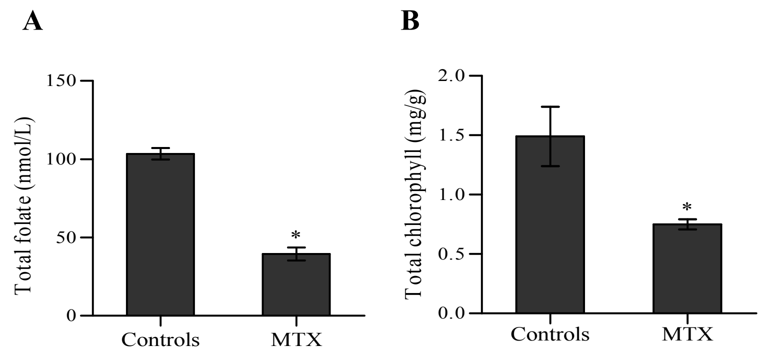

Figure S10. Effects of MTX treatment on the chlorophyll and total folate levels in leaves. The data are presented as the means ± SDs (n = 3). The statistical analysis was performed using one-way analysis of variance (ANOVA) followed by Duncan's multiple range test (DMRT) with three biological replicates. P-values ≤ 0.05 were considered significant.
